# Associations between telomere length, glucocorticoid receptor gene DNA methylation, volume of stress-related brain structures, and academic performance in middle-school-age children

**DOI:** 10.1016/j.cpnec.2023.100223

**Published:** 2023-12-24

**Authors:** Olga V. Burenkova, Oksana Yu. Naumova, Jessica A. Church, Jenifer Juranek, Jack M. Fletcher, Elena L. Grigorenko

**Affiliations:** a Department of Psychology, University of Houston, Houston, TX, United States; b Texas Institute for Measurement, Evaluation, and Statistics (TIMES), The University of Houston, Texas, United States; c Human Genetics Laboratory, Vavilov Institute of General Genetics RAS, Moscow, Russian Federation; d Department of Psychology, The University of Texas at Austin, Texas, United States; e Department of Pediatric Surgery at McGovern Medical School, The University of Texas Health Science Center at Houston, Texas, United States; f Center for Cognitive Sciences, Sirius University of Science and Technology, Sirius, Russian Federation

**Keywords:** HPA axis, DNA methylation, Telomere length, Immune cell composition, Brain, Academic performance

## Abstract

**Background:**

The biological embedding theory posits that early life experiences can lead to enduring physiological and molecular changes impacting various life outcomes, notably academic performance. Studying previously revealed and objective biomarkers of early life stress exposure, such as telomere length (TL), glucocorticoid receptor gene DNA methylation (DNAme), and the volume of brain structures involved in the regulation of HPA axis functioning (the hippocampus, the amygdala, and the medial prefrontal cortex), in relation to academic performance is crucial. This approach provides an objective measure that surpasses the limitations of self-reported early life adversity and reveals potential molecular and neurological targets for interventions to enhance academic outcomes.

**Methods:**

The participants were 52 children of Mexican or Central American origin aged 11.6–15.6 years. DNA methylation levels and TL were analyzed in three cell sources: saliva, whole blood, and T cells derived from whole blood.

**Results:**

Overall, the concordance across three systems of stress-related biomarkers (TL, DNAme, and the brain) was observed to some extent, although it was less pronounced than we expected; no consistency in different cell sources was revealed. Each of the academic domains that we studied was characterized by a unique and distinct complex of associations with biomarkers, both in terms of the type of biomarker, the directionality of the observed effects, and the cell source of biomarkers. Furthermore, there were biomarker-by-sex interaction effects in predicting academic performance measures.

**Conclusions:**

Assessed in an understudied youth sample, these preliminary data present new essential evidence for a deepened understanding of the biological mechanisms behind associations between exposure to early life stress and academic performance.

## Highlights

• The concordance across stress-related biomarkers (telomeric length, DNAme, and brain) is observed to some extent.
• No consistency in different cell sources (saliva, whole blood, and T cells) is registered.
• Each of the academic domains has a unique and distinct complex of associations with biomarkers.
• Biomarker-by-sex interaction affects predicting academic performance measures.

## 1 Introduction

According to the biological embedding theory, early life experiences can induce lasting physiological and molecular changes that become embedded in the biological systems, influencing such outcomes as health, wellbeing, learning, or behavior across the lifespan [1]. Academic performance holds a crucial position among these outcomes, as education is vital for empowering both personal wellbeing and societal progress by fostering a skilled workforce, reducing inequalities, and contributing to a globally competitive and stable society [[2], [3], [4]]. The most studied and widespread examples of biological embedding in humans are the physiological and molecular changes associated with exposure to early life stress (ELS), including adverse childhood experiences (ACEs). The latter have been demonstrated to be negatively associated with academic performance and related cognitive processes [[5], [6], [7], [8], [9]] providing a promising target for studying the interrelations between physiological and molecular changes and academic performance.

These physiological and molecular changes are underpinned by three major biological mechanisms: interrelated neurohumoral, genetic, and epigenetic processes [10], all of which are related to the functioning of the HPA (hypothalamic-pituitary-adrenal) axis, a neuroendocrine system crucial for the response to stress [11]. The expression of HPA axis activity can be divided into proximal, or hormonal (such as cortisol response, for example), and distal, which occur, at least in part, as a result of a change in the HPA axis activity pattern.

Among the distal mechanisms, the following changes have all been implicated as long-lasting outcomes of ELS exposure: alterations in brain structure and function (with the hippocampus, medial prefrontal cortex, and amygdala appear to be primary targets of exposure to stressors [[12], [13], [14], [15], [16]]), genomic changes, such as telomere shortening [17,18], and epigenetic alterations [19]. Since such distal biomarkers as neurobiological, genetic, and epigenetic ones are more stable than proximal/hormonal biomarkers against conditions preceding the sampling of biomaterial (such as circadian rhythms, consumption of food, liquids, and medication, physical activity/fitness, and sleep quality/quantity), assessing them can be more advantageous.

Studying these established biomarkers in relation to academic performance is important for at least two reasons. First, they serve as an objective proxy and cumulative outcome of ELS measures, which are self-reported in the vast majority of cases and, hence, biased. Moreover, these self-reported measures may hardly capture the entire complex picture of individual's life experiences. Second, in contrast to ELS measures, these objectively existing mechanisms can be targeted for interventions aimed at improving academic performance (for overview of some of these interventions, see McEwen [20]). For these two reasons we specifically aimed to study the association between markers of biologically embedded ELS ‒ telomere length, glucocorticoid receptor gene DNA methylation, and the volume of stress-related brain structures ‒ and measures of academic performance.

It is important to note here that the measures of biomarkers such as telomere length (TL) and DNAme levels depend on the cell composition of the tissue used as a DNA source since both TL [[21], [22], [23]] and DNAme [[24], [25], [26]] are tissue/cell-specific, owing to different rates of cell division [21,27] and the epigenetic profiles [28] of distinct cell types. In addition, chronic and transient stress could induce alteration in the immune cell composition in the blood [[29], [30], [31]]. Therefore, the measures of biomarkers such as TL and DNAme in complex tissues could depend on both direct changes of these measures in response to exposure to stressors and also on stress-dependent changes in the immune cell composition. Thus, immune cell composition should be taken into account when studying TL and DNAme levels.

Despite the importance of studying the interplay between markers of ELS exposure and academic underperformance, data on the concordance between the abovementioned biological markers and their relations with academic performance are still scarce or, more commonly, even absent, especially in the pediatric population. In this pilot study, we report the associations between three pre-selected distal biomarkers of ELS (brain-based, epigenetic, and telomeric) and academic performance within the sample of middle-school-aged children. We hypothesized that shorter TL, a higher level of DNAme in the glucocorticoid receptor (GR*)* gene (*NR3C1*), and lower volumes of the hippocampus, amygdala, and medial prefrontal cortex would be associated with each other and with lower academic performance. Notably, our work involves several biomarkers at once, explores their understudied interrelations, and specifically focuses on their associations with critical cognitive outcomes and children's academic performance. Moreover, we test the consistency of these epigenetic and telomeric associations across three types of peripheral cells and tissues (saliva, blood, and peripheral T lymphocytes) commonly used as DNA sources in behavior genetics studies.

## 2 Methods and materials

### 2.1 Participants

This pilot study included 52 children (21 girls and 31 boys) of Mexican or Central American origin from low-income families aged between 11.6 and 15.6 years (M±SD = 12.65 ± 0.83 years of age) who had no history of severe developmental issues or physical and mental health problems. Spanish was the home language for these children. All adolescents were sufficiently proficient to receive classroom instruction in English as determined by the state assessment of English language proficiency, the Texas English Language Proficiency Assessment System (TELPAS; https://www.texasassessment.gov/telpas.html). Students who were recent immigrants or who did not demonstrate the proficiency needed on the TELPAS for a full transition to English were excluded from the study. These 6th-8th graders were recruited as part of a larger study of reading ability by the Texas Center for Learning Disabilities. We limited the recruitment only to adolescents of Mexican/Central American descent to reduce heterogeneity because the variation in ancestry can affect epigenetics and other markers of stress exposure as well as underlying racial health disparities between human populations [32]. We were limited in the number of participants who could be included in the current study due to the requirement of obtaining both MRI data and blood samples, which could only be collected during the MRI session with the assistance of a phlebotomist. However, saliva samples were available from a larger number of children.

Forty-three of 52 (83 %) participants provided information on the total pre-tax household income over the past year. When dividing the sample by median income, we did not reveal between-group differences in any biological or academic indicators; no statistically significant correlations between income and any biological or academic indicators were found either. For this reason, we did not consider the SES measure in our analyses.

The study was approved by the University of Houston Institutional Review Board (IRB Reference Number STUDY00000554) in compliance with the Declaration of Helsinki; all guardians signed informed consent, and all participants signed assent.

### 2.2 Samples collection and DNA extraction

Blood (whole blood, WB) and saliva samples were collected at the time of the participants' MRI visits. Saliva was self-collected into the Oragene-DNA tubes (DNA Genotek, Ottawa). Salivary DNA was isolated using the prepIT L2P reagent according to the manufacturer's instruction (DNA Genotek, Ottawa), followed by DNA purification and concentration using the Genomic DNA Clean and Concentrator kit (Zymo Research Corporation, Irvine, CA, USA). Venous blood was collected into EDTA anticoagulant lavender top vacutainer tubes and was transferred on ice to the lab as soon as possible. Extraction of genomic DNA from the WB was performed using the Flexi Gene DNA kit according to the manufacturer's protocol (Qiagen, Germany). CD3^+^ cells (T cells) were isolated from WB using CD3 magnetic beads, accompanying reagents, and the MultiMACS Cell24 separator Plus (all from Miltenyi Biotec, Bergisch-Gladbach, Germany). The Allprep DNA/RNA kit (Qiagen, Germany) was then utilized for DNA extraction from CD3^+^ cells according to the manufacturer's instructions.

### 2.3 DNA methylation profiling

Whole-genome DNAme profiling was performed using the Illumina MethylationEPIC assay, which interrogates over 850,000 CpG sites across the human genome regulatory regions. The DNA bisulfite treatment, hybridization on the array, and the BeadChip scanning using the Illumina iScan were performed according to the manufacturer's instructions (Illumina, San Diego, CA, USA). For the microarray data processing, the Minfi R package was used [33]. The probes with detection p-values greater than 0.05 and probes with missing values were removed from further analyses. The stratified quantile normalization implemented by the preprocessQuantile function in the *minfi* package was applied for the data normalization. *Minfi* was used to calculate methylation levels (beta-values), where beta-values represent the ratio of methylated to total signal, spanning from 0 (indicating complete unmethylation) to 1 (indicating complete methylation).

DNAme levels at five loci at 1F exon and promoter of the GR gene (cg17860381, cg04111177, cg15910486, cg15645634, cg18068240) were derived from the whole genome methylation panel and included in this study. In addition, estimation of the six different white blood cell types (CD8^+^ T and CD4^+^ T lymphocytes, CD56^+^ natural killer cells, CD19^+^ B cells, CD14^+^ monocytes, and granulocyte neutrophils) was performed by applying the ‘estimateCellCounts’ function of the Minfi package that utilizes an algorithm developed to estimate immune cell composition based on DNA methylation profiles [34] with FlowSorted.Blood.EPIC is used as the underlying reference dataset. Descriptive statistics for the immune cell composition are presented in Table 1.

**Table 1:** Descriptive statistics for the proportion of immune cells of each type.

	CD8+T lymphocytes	CD4^+^ T lymphocytes	CD56^+^ natural killer cells	CD19+B cells	CD14^+^ monocytes	Granulocyte neutrophils
	Mean ± SD, [min-max]					
Saliva	0.04 ± 0.02, [0–0.1]	0.11 ± 0.03, [0.02–0.19]	0.01 ± 0.01, [0–0.04]	0.06 ± 0.02, [0.01–0.13]	0.09 ± 0.04, [0.01–0.18]	0.75 ± 0.10, [0.50–0.96]
T cells	0.38 ± 0.08, [0.23–0.59]	0.47 ± 0.09, [0.23–0.64]	0.07 ± 0.04, [0–0.2]	0.03 ± 0.03, [0–0.15]	0.01 ± 0.01, [0–0.05]	0.03 ± 0.03, [0–0.2]
WB	0.13 ± 0.04, [0.05–0.23]	0.11 ± 0.04, [0.02–0.21]	0.06 ± 0.03, [0–0.15]	0.08 ± 0.03, [0.02–0.15]	0.07 ± 0.02, [0.03–0.14]	0.55 ± 0.09, [0.35–0.71]

We regressed DNAme measures on the cell-type count for the purpose of the reduction of the number of variables and, at the same time, for controlling for immune cell composition, taking into account a high level of correlations between the immune cell-type composition and DNAme levels (Table A1). The standardized residual values were used for further analyses.

### 2.4 Telomere length

TL was determined in triplicate using the q-PCR method described by O'Callaghan and Fenech [35] to measure absolute TL. Real-Time PCR assays were performed using a QuantStudio 7 Flex Real-Time PCR System (Applied Biosystems, USA). The *36B4* gene (acidic ribosomal phosphoprotein) was used as a single-copy gene (S) to normalize DNA input. The following primers were used: 36B4F (CAGCAAGTGGGAAGGTGTAATCC) and 36B4R (CCCATTCTATCATCAACGGGTACAA) at a final concentration of 0.1 μM. TL was measured using the following primers: teloF (CGGTTTGTTTGGGTTTGGGTTTGGGTTTGGGTTTGGGTT) and teloR (GGCTTGCCTTACCCTTACCCTTACCCTTACCCTTACCCT), both at a final concentration of 0.1 μM. All primers and oligomer standards were purchased from Eurofins Genomics (Louisville, KY). For each PCR reaction, 10 μL of PowerUp™ SYBR™ Green Master Mix (2x) (Thermo Fisher Scientific, USA) and 20 ng of DNA were used, in a total volume of 20 μl per well. A standard curve was generated on each 96-well plate by performing a serial dilution of a known amount of oligomer standard for either 36B4 or telomere. Plasmid DNA (pBR322, Sigma) is added to each standard to maintain a constant 20 ng of total DNA per reaction tube.

The thermal cycling PCR reaction was initiated with a 95C incubation for 10 min, followed by 40 cycles of 95C for 15 s and 60C for 1 min, followed by a dissociation (melt) curve. The efficacy level for all reactions fell within the range of 90–110 %. No samples with a difference in Ct value of >1 between samples in triplicate were excluded. In total, 8 plates for telomere and 8 plates for 36B4 were run. Absolute TL (kb per diploid genome) in each sample resulted from the average triplicate of TL in kb divided by the average triplicate of the number of diploid genomes (36B4). Interquartile mean (IQM) normalization of data was performed to eliminate between-plate variability [36].

We regressed TL measures on the cell-type count for the purpose of the reduction of the number of variables and, at the same time, for controlling for immune cell composition, taking into account a high level of correlations between the immune cell-type composition and TL levels (Table A1). The standardized residual values were used for further analyses.

### 2.5 Brain imaging data

The MRI data were acquired using a Siemens 3T scanner at two locations: Austin (Vida, n = 23) and Houston (Prisma scanner, n = 29). Adolescent Brain Cognitive Development (ABCD) study protocol [37] was used. High-resolution anatomical images covering the entire brain were obtained with accelerated 3D T1-weighted and 3D T2-weighted sequences (matched geometry) for surface-based analyses of cortical volume using Freesurfer v7.1.0 software. Isotropic images (0.8*0.8*0.8 mm^3^) were acquired in the sagittal plane with FoV = 256*256, 3D-T1: TR/TE = 2400/2.22 ms, α = 8°. 3D-T2: TR/TE = 3200/563 ms. Regions of interests (ROIs) were labeled using the Desikan-Killany atlas [38] (2 hemispheres): volume of the hippocampus, the amygdala, and the medial prefrontal cortex (mPFC) corrected for estimated intracranial volume. Because measures from the right and left parts of each brain area were highly correlated (Table A2), we averaged z scores across hemispheres to reduce the number of variables.

### 2.6 Academic performance

The tests of academic performance administered in school and used in this study are presented in Table 2. We used raw instead of age-based standard scores as correction for age was carried out at the statistical analysis stage (see below). A reading composite score was created by averaging the KTEA-3 Letter & Word Recognition, KTEA-3 Word Recognition Fluency, and GMRT Comprehension z scores. Similarly, a math composite score was computed as the average of a subject's KTEA-3 Math Computation and KTEA-3 Math Fluency z scores. Finally, a writing composite score was created by averaging the TOWL-4 Contextual Conventions and TOWL-4 Story Composition z scores.

**Table 2:** Corresponding of tests to domains and constructs.

Domain	Test	Subtests	Construct	Reference
Reading (composite score)	Kaufman Test of Educational Achievement, Third Edition (KTEA-3)	Letter & Word Recognition	Decoding	Kaufman and Kaufman [39]
		Word Recognition Fluency	Fluency	
	Gates-MacGinitie Reading Tests' Comprehension subtest (GMRT)	Comprehension	Comprehension	MacGinitie [40]
Math (composite score)	Kaufman Test of Educational Achievement, Third Edition (KTEA-3)	Math Computation	Calculations	Kaufman and Kaufman [39]
		Math Fluency	Fluency	
Writing (composite score)	Test of Written Language, Fourth Edition (TOWL-4)	Contextual Conventions	Written Expression	Hammill and Larsen [41]
		Story Composition	Written Expression	
Language	Woodcock-Johnson Tests of Cognitive Abilities-Third Edition (WJ III)	Picture Vocabulary	Vocabulary	Woodcock et al. [42]
IQ Estimate	Wechsler Abbreviated Scale of Intelligence, Second Edition (WASI-II)	Matrix Reasoning	Intelligence	Wechsler [43]

### 2.7 Statistical analysis

Median imputation was used to handle missing data (TL = 3.2 %; each CG = 0.6 %; KTEA-3 Letter & Word Recognition, Word Recognition Fluency, Math Computation, Math Fluency, and GMRT Comprehension = 1.9 % each).

*Spearman's partial correlations* were used to analyze associations between biological and academic measures since not all data were normally distributed. Jamovi statistical software, version 1.6.23.0 [44], was utilized for this purpose. All correlations were corrected for age and sex. The Bonferroni-Holm adjustment for multiple pairwise comparisons [45] was performed using WinPepi [46]. Bonferroni-Holm adjustment is less conservative than the original Bonferroni method and is acceptable for both parametric and non-parametric analyses. In addition to the correlation analysis aimed at defining the relationship between various biomarkers as well as between single biomarkers and single academic performance measures in a pairwise manner, we also performed regression analysis across biomarkers to study the cumulative contribution of sets of biomarkers in predicting individual academic outcomes.

*General linear models* (GLM) were utilized to estimate the effects of biological measures, age, sex, and interaction of all biological measures and age with sex on each academic performance measure. The age variable was centered around its mean.

For each of the five academic performance measures and each of the three cell sources, we searched for the best model. For this purpose, for each of these 15 models, we evaluated the subset of candidate models with all combinations of all biomarkers, sex, and age as predictors and one outcome (academic performance measure) at a time. A model selection procedure based on Akaike's information criterion (AIC) corrected for a small sample size AICc, Burnham and Anderson [47] in the R package MuMIn [48] was used for this evaluation: from each subset, the model with minimal ΔAICc was considered the best. The resulting 15 best models are presented in Table A3.

Then the statistical significance of each best model was examined by the F-test followed by the *t*-test using the lm R function (Version 4.1.2; R Development Core Team [49]. Model diagnostics included testing for multicollinearity, homogeneity of the residuals with Q-Q plots (heteroscedasticity), and normal distribution of residuals with the Shapiro-Wilk test by means of R package olsrr [50]. The outliers were diagnosed by checking Cook's distance values. No observations with a Cook's distance greater than 1 were revealed, and thus, none were excluded from the regression analyses.

In all analyses, the threshold of significance level was a corrected p ≤ .05.

## 3 Results

### 3.1 Concordance between biological markers

First, we tested the strength of relations among the biomarkers (brain, TL, and DNAme), taking into account cell sources (saliva, T cells, and WB). After correction for multiple testing, the only significant result was a positive correlation between TL in saliva and DNAme level at cg15645634 (Table 3). Among other results that did not survive correction for multiple testing, the following patterns were observed: 1) positive correlation between TL in T cells and the volume of the amygdala; 2) positive correlation between DNAme level at cg15910486 and TL in T cells. The largest number of suggestive associations were found in T cells, followed by saliva, and no suggestive correlations were found in WB.

**Table 3:** Spearman's rho correlation coefficients for associations between biological markers.

	TL	cg17860381	cg04111177	cg15910486	cg15645634	cg18068240
**Saliva**						
TL	–	−0.13	−0.04	0.10	−0.37**	−0.01
Hippocampus volume	0.20	−0.20	−0.17	−0.15	−0.09	0.05
Amygdala volume	0.22	0.11	−0.01	0.01	−0.22	−0.28
mPFC volume	−0.05	−0.18	0.09	0.23	−0.02	−0.21
**T cells**						
TL	–	0.14	−0.05	0.35*	−0.02	−0.12
Hippocampus volume	0.08	0.10	−0.13	0.14	−0.07	−0.04
Amygdala volume	0.30*	−0.08	0.09	0.05	−0.10	−0.07
mPFC volume	0.01	−0.04	−0.01	−0.08	−0.16	−0.27
**WB**						
TL	–	−0.13	0.03	0.17	−0.13	−0.24
Hippocampus volume	−0.09	−0.03	−0.09	−0.16	0.09	0.06
Amygdala volume	0.17	−0.19	0.17	−0.12	−0.03	−0.04
mPFC volume	−0.11	0.01	0.16	−0.18	0.03	0.10

*Note.* controlling for age and sex.

*p < .05, **p < .01 not adjusted for multiple comparisons. The associations survived after correction for multiple testing are shaded gray (p < .05).

### 3.2 Associations between biological markers and academic performance

Next, we addressed our key question of how these different biomarkers are related to academic performance. No results survived correction for multiple testing; however, the following patterns were observed (Table 4): 1) both salivary and blood TL were negatively correlated with language performance (WJ III: Picture Vocabulary); 2) negative correlations between DNAme levels and academic performance levels were revealed for two loci in WB (cg15645634 with Writing composite score and WJ III: Picture Vocabulary; cg18068240 with Writing composite score); 3) both hippocampal and amygdalar volumes were negatively correlated with Writing composite score. The largest number of suggestive correlations was found in WB saliva, followed by saliva, and no correlations were found in T cells.

**Table 4:** Spearman's rho correlation coefficients for associations between biological markers and academic performance.

	TL	cg17860381	cg04111177	cg15910486	cg15645634	cg18068240	Hippocampus volume	Amygdala volume	mPFC volume
**Saliva**									
Reading composite score	−0.19	−0.07	0.15	0.23	0.26	0.04	−0.08	−0.06	0.09
Math composite score	−0.17	−0.18	−0.05	0.15	0.12	−0.01	−0.09	0.00	0.05
Writing composite score	−0.01	0.17	0.06	0.23	0.15	−0.04	−0.39**	−0.28*	−0.08
WJ III: Picture Vocabulary	−0.29*	0.11	0.05	0.17	0.16	−0.13	−0.25	0.02	0.12
WASI-II Matrix Reasoning	−0.2	0.12	0.17	0.2	−0.03	−0.04	−0.16	−0.11	−0.09
**T cells**									
Reading composite score	0.08	−0.15	−0.08	−0.03	0.06	−0.05			
Math composite score	−0.13	−0.04	−0.14	0.06	−0.22	−0.27			
Writing composite score	0.07	−0.11	−0.12	0.01	0.07	−0.13			
WJ III: Picture Vocabulary	−0.06	−0.03	−0.12	−0.08	0.06	−0.07			
WASI-II Matrix Reasoning	−0.07	−0.06	−0.11	−0.05	−0.07	−0.12			
**Whole Blood**									
Reading composite score	−0.12	−0.1	0.07	−0.17	−0.15	−0.15			
Math composite score	−0.16	−0.04	−0.09	0.05	−0.2	−0.19			
Writing composite score	0.02	0.13	−0.13	0.25	−0.32*	−0.28*			
WJ III: Picture Vocabulary	−0.29*	0.03	0.09	−0.16	−0.29*	−0.18			
WASI-II Matrix Reasoning	−0.11	−0.05	−0.24	0.05	−0.22	−0.19			

*Note.* controlling for age and sex.

*p < .05, **p < .01 not adjusted for multiple comparisons. No associations survived after correction for multiple testing.

### 3.3 Contribution of biomarkers to academic performance

Of 15 candidate GLM models (for each of 3 cell sources and 5 academic performance measures, Table A3), 6 models describing the association between biomarkers and academic performance were both statistically significant and passed model diagnostics (Table 5).

**Table 5:** General linear models for academic performance measures as a function of biological measures, age, sex, and interaction of biological measures and age with sex.

	Model 1			Model 2			Model 3		
	Reading composite score			Writing composite score			Writing composite score		
	B	SE B	β	B	SE B	β	B	SE B	β
Constant	0.00	0.11		−0.29	0.14		−0.27	0.15	
Sex(female)				0.70	0.23	0.38**	0.62	0.23	0.34*
TL_whole blood				−0.28	0.12	−0.29*			
cg15910486_saliva	0.28	0.13	0.31*						
cg15645634_saliva	0.30	0.13	0.33*						
cg04111177_whole blood				0.36	0.16	0.37*			
Volume of Hippocampus				−0.40	0.15	−0.38**	−0.35	0.14	−0.34*
Volume of Amygdala				0.29	0.15	0.30			
Volume of mPFC							0.60	0.21	0.54**
cg04111177_whole blood * Sex				−0.61	0.23	−0.43*			
Volume of mPFC * Sex							−0.64	0.28	−0.40*
F for Model	3.96*			4.12**			4.27**		
Adjusted R^2^	0.10			0.27			0.20		

	Model 4			Model 5			Model 6		
	*WJ III: Picture Vocabulary*			*WJ III: Picture Vocabulary*			*WJ III: Picture Vocabulary*		
	B	SE B	β	B	SE B	β	B	SE B	β
Constant	0.18	0.16		0.14	0.16		0.16	0.15	
Sex(female)	−0.50	0.26	−0.25	−0.41	0.25	−0.20	−0.51	0.24	−0.25*
TL_whole blood							0.09	0.19	0.08
cg15910486_saliva	0.22	0.14	0.20						
cg15645634_ t cells				0.31	0.18	0.30			
cg18068240_ t cells				−0.46	0.18	−0.43*			
cg15645634_ whole blood							−0.39	0.13	−0.37**
Volume of Hippocampus	−0.57	0.20	−0.49**	−0.58	0.19	−0.50**	−0.56	0.19	−0.49**
Volume of Hippocampus * Sex	0.54	0.30	0.31	0.56	0.29	0.32	0.69	0.28	0.39*
TL_whole blood * Sex							−0.53	0.26	−0.37*
F for Model	3.79**			4.04**			4.55**		
Adjusted R^2^	0.18			0.23			0.30		

Note. *p < .05, **p < .01.

In Model 1, DNAme levels at cg15910486 and cg15645634 in saliva explained 10 % of the variance in the Reading composite score. Higher DNAme levels at both loci were significantly associated with higher Reading composite scores.

Variance in the Writing composite score was explained by two sets of biomarkers. In Model 2, sex, TL, and DNAme at cg04111177 in WB, the interaction between WB DNAme at cg04111177 and sex and the volumes of the hippocampus and amygdala explained 27 % of the variance in the Writing composite score. Lower TL in WB, higher DNAme level at cg04111177 in WB, lower volume of the hippocampus, and female sex were significantly associated with higher Writing composite scores. Furthermore, DNAme level at cg04111177 in WB significantly interacted with sex in predicting this academic measure. Particularly, a negative slope was observed in females (i.e., the Writing composite score decreased as the DNAme level increased), whereas the slope in males was positive.

In Model 3, sex, the volumes of the hippocampus and mPFC, and the interaction between hippocampal volume and sex explained 20 % of the variance in the Writing composite score. Like in the previous model, lower volume of the hippocampus and female sex were significantly associated with a higher Writing composite score. However, the higher volume of mPFC was significantly associated with a higher Writing composite score. Furthermore, hippocampal volume significantly interacted with sex in predicting this academic measure: specifically, a negative slope was observed in females (i.e., the Writing composite score decreased as the hippocampal volume increased), whereas the slope in males was positive.

Variance in the Language domain (WJ III Picture Vocabulary) was explained by three sets of biomarkers. In Model 4, sex, DNAme levels at cg15910486 in saliva, the volume of the hippocampus, and its interaction with sex explained 18 % of the variance in this academic score. A lower volume of the hippocampus was significantly associated with higher WJ III Picture Vocabulary scores. In Model 5, sex, DNAme levels at cg15645634 and cg18068240 in T cells, the volume of the hippocampus, and its interaction with sex explained 23 % of the variance in this academic score. Again, a lower volume of the hippocampus was significantly associated with higher WJ III Picture Vocabulary scores, as well as lower DNAme levels at cg18068240 in T cells. Finally, in Model 6, sex, TL in WB and its interaction with sex, DNAme levels at cg15645634 in WB, the volume of the hippocampus and its interaction with sex explained 30 % of the variance in this academic score of the Language domain. Lower volume of the hippocampus, male sex, and lower DNAme levels at cg15645634 in WB was significantly associated with higher WJ III Picture Vocabulary scores. Furthermore, hippocampal volume significantly interacted with sex in predicting this academic measure: particularly, a steeper negative (i.e., this score decreased as the hippocampal volume increased) slope was revealed in males than in females. TL in WB also interacted with sex: a negative slope was observed in females, whereas the slope in males was positive.

## 4 Discussion

The aim of this research was twofold: (1) to explore the concordance across three systems of stress-related biomarkers – the brain structural characteristics, TL, and the GR gene DNAme levels measured in three various types of peripheral tissues and cells (saliva, WB, T cells), and (2) to examine the association of these biomarkers with academic performance in children. Among studied biomarkers, TL was the only biomarker significantly associated with other biomarkers that survived correction for multiple comparisons. Specifically, TL negatively correlated with DNAme levels at cg15645634 in saliva, which is consistent with our hypothesis regarding a negative association between TL and GR gene methylation. At the moment, there seems to be no additional empirical evidence to verify any of these observations, and hence the relationship observed here in middle-school-age children could be regarded as a starting point.

Speaking about the associations between TL and volumetric characteristics of the brain, TL in T cells showed a suggestive association (positive correlation) with the amygdala volume, which is consistent with our hypothesis regarding the positive relationship of TL with brain volume measures. Importantly, this relationship was observed in middle-school-age children, filling a gap in the literature. Previous data, which showed the same pattern of a positive association between TL and hippocampal volume, were obtained in a sample of adults [51,52].

We have not revealed any relationship between DNAme levels and brain indexes. The literature confirms the absence of a consistent relationship between DNAme levels and brain measures, albeit in adults, as no significant association between DNAme levels at the GR gene promoter in the peripheral blood and hippocampal volume was registered [53]. According to our knowledge, such associations have never been solicited in a pediatric sample.

Overall, the concordance across three systems of stress-related biomarkers (TL, DNAme, and brain) took place to some extent, although it was less pronounced than we expected. Importantly, we did not observe the consistency of these results in different cell sources; for example, no associations between biomarkers were found in WB, in contrast to saliva and T cells, where some were detected. It is hard to juxtapose this finding with the data from the three previously mentioned reports [[51], [52], [53]] as none of them was performed on saliva, and none of them utilized the procedure of controlling for immune cell composition. In the light biological embedding theory, these results suggest a complex interplay among ELS-related biomarkers. The study offers nuanced insights into the relationships among these biomarkers and underscores the necessity for a more comprehensive understanding of the biological embedding process.

To test the associations between biomarkers and academic performance, we used two approaches: correlation analysis between biomarkers and academic performance and the examination of the contribution of a set of significant biomarkers to each academic domain. For two domains, IQ and Math, we observed the absence of the impact of biomarkers and such biological factors as sex and age. For other domains, the registered associations were shown to be both positive and negative, as discussed below.

TL was negatively associated with both performance in the Writing (WB only) and Language domains (WB and saliva), based on suggestive correlations; regression analysis revealed the same statistically significant pattern for TL in WB and performance in the Writing domain. This was observed for the first time in the pediatric sample and contradicted our initial hypothesis, derived from the adult literature [[54], [55], [56]], suggesting that longer TL might be associated with higher academic performance. However, it is important to note that none of these authors applied the procedure of controlling for immune cell composition, and these methodological differences may account for the discrepancies in the results.

Regarding the association between the GR gene methylation and academic performance, we observed a suggestive negative correlation between DNAme levels at cg15645634 in WB and measures of the Writing (along with DNAme levels at cg18068240 in WB) and Language domains. Based on regression analysis, DNAme levels at cg15645634 in WB were as well negatively associated with performance in the Language domain, along with DNAme levels at cg18068240 in T cells. This finding confirmed our hypothesis that a lower level of DNAme might be associated with higher academic performance. The only supporting literature data showed a significant positive association between placental DNAme levels of the GR gene and the odds of developing moderate/severe adverse cognitive impairment in children at age 10; this sample did not include the component of stress exposure [57]. However, DNAme levels in saliva, both at cg15910486 and cg15645634, were positively associated with the Reading composite, as well as DNAme levels at cg04111177 in WB were positively associated with the Writing composite based on regression analysis, reflecting, perhaps, that different cell sources and DNAme loci may play opposite roles in the association between ELS exposure and academic performance.

The volume of mPFC was positively associated, according to regression analysis, with the Writing domain; this confirmed our hypothesis and was in line with existing literature [16]. In contrast, the volume of the hippocampus was negatively associated with both the Writing and Language domains, based on regression analysis, and with the Writing domain based on suggestive correlation. According to the literature derived from children's samples, bilateral anterior hippocampal volumes positively predict scores in the picture vocabulary task of the NIH Toolbox for the Assessment of Neurological and Behavioral Function Cognition Battery [58]; positive correlations were observed between right hippocampal volume and language expressive and receptive scores of the Mullen Scales of Early Development [59]. It is possible that the relationship between hippocampal volume and language performance may go beyond the proposed mechanisms of the effect of ELS exposure on cognitive function. The volume of the amygdala was negatively associated with the Writing domain as well, according to suggestive correlation. However, the data on the relationship between the volume of the amygdala and ACEs exposure are contradictory and point to both an increase [14,15] and a decrease [12,16] in its volume.

In total, each of the academic domains we studied was characterized by a unique and distinct complex of associations with biomarkers in terms of their directionality, the type of biomarker, and the cell source of biomarkers. The diversity of these associations ranged from absence, as observed for IQ and Math, to associations with all types of studied biomarkers in all cell sources, with the exception of T cells, as for Writing and Language. Regarding our initial hypotheses, in general, it should be noted that the real picture is more complicated than we expected, according to the theoretical premises and the limited empirical literature. In fact, we seem to be dealing with an ensemble of complexly organized and interconnected associations, depending, among other things, on the participants’ sex. In the literature cited here, the effect of sex was not investigated, except for one report [59]. The authors showed that left hippocampal volume in children aged 2–4 years positively predicted receptive language in females but not males, which somewhat corresponded to our data where a steeper negative slope of the regression line between hippocampal volume and Language ability measure was more prominent in males than in females.

An important observation is that across all cell sources that we analyzed, despite adjusting for immune cell composition, various directions of associations of biomarkers with academic performance indicators were observed. Given that DNAme changes in peripheral tissues “are not surrogates of brain changes but reflect a coordinated brain-body system-wide response to experience and exposure derived challenges” [60], this observation might reflect the particular role of each tissue in response to the environmental challenge. The same could probably be relevant to TL derived from different cell sources. Moreover, it is possible that the longitudinal relationship between the TL attrition rate and the dynamics of cognitive performance [61] rather than the analysis of the static association of TL and cognitive performance might be informative in the context of the association between stress biomarkers and academic achievement. In the context of the biological embedding theory, the findings regarding the associations between stress biomarkers and academic performance highlight the interconnectedness between physiological and molecular changes embedded in biological systems and cognitive outcomes.

The study is characterized by a number of limitations. First, our sample was low-income (the median household income in our sample was $30,000 compared to the median household income in Texas of $63,826 [62] and from low-performing schools relative to other youth in Texas. Next, as a modest sample size (n = 52) was used, further investigations with a larger sample size will be required. Further, the results of this study were obtained from a sample of children of Mexican or Central American origin and require replication in other ancestry cohorts. Also, since these results were derived from cross-sectional and not longitudinal observations, no direct inference about the causal impact that biomarkers may have on academic performance can be made. Another important consideration is that, although less conservative than the original Bonferroni method, the utilized correction approach is still a rather conservative method of adjustment for multiple comparisons, potentially eliminating some important findings. In addition, the use of computer simulation techniques aimed to reveal the correct decision rates for multiple comparison procedures was not possible here due to the small sample size.

To validate the observed biomarker associations, considering the preliminary nature of the current pilot study, it is vital to conduct replication studies. In these studies, the implementation of the following advancements would be extremely advantageous: 1) expanding the sample size to accommodate the utilization of multiple biological and academic markers, thereby increasing the study's power; 2) diversifying the population to enhance the generalizability of findings in terms of ethnic composition; 3) taking into account such covariates as BMI, pubertal development, substance use, the rate of foreign-born versus native-born youth, and the average duration of residence in the new country for foreign-born youth; and 4) implementing a longitudinal design to better capture the dynamic nature of the relationships between stress biomarkers and academic performance over time.

In conclusion, the preliminary findings of this pilot study, guided by the biological embedding theory, offer insights into the complex relationship between ELS biomarkers and academic performance in a youth sample of Mexican or Central American origin. The identification of unique associations across different biomarkers, academic domains, and cell sources highlights the complexity of this interplay. By examining objective biomarkers such as telomere length, glucocorticoid receptor gene DNA methylation, and the volume of brain structures involved in the regulation of HPA axis functioning, the study provides an objective estimate that surpasses the limitations of self-reported ELS. Moving forward, this work lays the groundwork for larger-scale investigations that can enhance our understanding of potential biological mechanisms underlying academic underperformance and promote the development of interventions targeting these mechanisms.

## CRediT authorship contribution statement

**Olga V. Burenkova:** Conceptualization, Formal analysis, Writing – original draft, Writing – review & editing. **Oksana Yu. Naumova:** Conceptualization, Data curation, Funding acquisition, Project administration, Supervision. **Jessica A. Church:** Conceptualization, Data curation, Funding acquisition, Project administration, Supervision. **Jenifer Juranek:** Conceptualization, Data curation, Funding acquisition, Project administration, Supervision. **Jack M. Fletcher:** Conceptualization, Funding acquisition, Project administration, Supervision. **Elena L. Grigorenko:** Conceptualization, Funding acquisition, Project administration, Supervision.

## Declaration of competing interest

None.
