# Supplementary material for: Associations between telomere length, glucocorticoid receptor gene DNA methylation, volume of stress-related brain structures, and academic performance in middle-school-age children
Source: Compr Psychoneuroendocrinol. 2023 Dec 24;17:100223. doi: 10.1016/j.cpnec.2023.100223 (PMC10787263; doi:10.1016/j.cpnec.2023.100223)
Supplement: Multimedia component 1 [file mmc1.docx]

**Appendix A**

**Associations between telomere length, glucocorticoid receptor gene DNA methylation, volume of stress-related brain structures, and academic performance in middle-school-age children**

*Burenkova et al.*

Table A1. Spearman’s rho correlation coefficients for associations between immune cell composition, TL and DNAme levels

|  | TL | cg17860381 | cg04111177 | cg15910486 | cg15645634 | cg18068240 |
| --- | --- | --- | --- | --- | --- | --- |
| CD8+ T lymphocytes | -0.64*** | -0.26*** | -0.34*** | -0.63*** | 0.25** | 0.26** |
| CD4+T lymphocytes | -0.53*** | -0.14 | -0.25** | -0.41*** | 0.16* | 0.18* |
| CD56+natural killer cells | -0.50*** | -0.24** | -0.26** | -0.63*** | 0.19* | 0.20* |
| CD19+B cells | 0.28*** | 0.24** | 0.05 | 0.18* | -0.04 | -0.02 |
| CD14+monocytes | 0.48*** | 0.29*** | 0.24** | 0.47*** | -0.24** | -0.25** |
| Granulocyte neutrophils | 0.65*** | 0.29*** | 0.32*** | 0.66*** | -0.28*** | -0.29*** |
| *Note.* controlling for age and sex  * p < .05, ** p < .01, *** p < .001 not adjusted for multiple comparisons. The associations survived after correction for multiple testing are shaded gray (p < .05). | | | | | | |

Table A2. Spearman’s rho correlation coefficients for associations between left and right hemispheres

|  | R Hippocampus volume | R Amygdala volume | R mPFC volume |
| --- | --- | --- | --- |
| L Hippocampus volume | 0.49*** |  |  |
| L Amygdala volume |  | 0.76*** |  |
| L mPFC volume |  |  | 0.29* |
| *Note.* controlling for age and sex  * p < .05, *** p < .001 | | | |

Table A3. Best model selection results describing the effects of biological measures, age, sex, and interaction of biological measures and age with sex on academic performance measures.

| Cell source | Model | | df | logLik | AICc | | ΔAICc | AICc weight |
| --- | --- | --- | --- | --- | --- | --- | --- | --- |
|  | Outcome | Predictor |  |  |  | |  |  |
| Saliva | *Reading composite score* | |  |  |  | |  |  |
|  | **cg15645634 + cg15910486** | | 4 | -61.2 | 131.3 | | 0 | 0.35 |
|  | *Math composite score* | |  |  |  | |  |  |
|  | **cg15645634 + cg15910486 + cg17860381** | | 5 | -61.4 | 134.1 | | 0 | 0.13 |
|  | *Writing composite score* | |  |  |  | |  |  |
|  | **Volume of Hippocampus + Volume of mPFC + Volume of mPFC*Sex + Sex(female)** | | 6 | -60.1 | 134.1 | | 0 | 0.02 |
|  | *WJ III: Picture Vocabulary* | |  |  |  | |  |  |
|  | **cg15910486 +Volume of Hippocampus + Volume of Hippocampus*Sex(female) + Sex(female)** | | 6 | -66.0 | 145.9 | | 0 | 0.09 |
|  | *WASI-II Matrix Reasoning* | |  |  |  | |  |  |
|  | **TL** | | 3 | -71.5 | 149.5 | | 0 | 0.24 |
| T cells | *Reading composite score* | |  |  |  | |  |  |
|  | **Age** | | 3 | -64.5 | 135.6 | | 1.06 | 0.18 |
|  | *Math composite score* | |  |  |  | |  |  |
|  | **cg18068240 + Age** | | 4 | -62.4 | 133.7 | | 0 | 0.12 |
|  | *Writing composite score* | |  |  |  | |  |  |
|  | **Volume of Hippocampus + Volume of mPFC + Volume of mPFC*Sex(female) + Sex(female)** | | 6 | -60.1 | 134.1 | | 0 | 0.48 |
|  | *WJ III: Picture Vocabulary* | |  |  |  | |  |  |
|  | **cg15645634 + cg18068240 + Volume of Hippocampus + Volume of Hippocampus*Sex(female) + Sex(female)** | | 7 | -63.8 | 144.2 | | 0 | 0.14 |
|  | *WASI-II Matrix Reasoning* | |  |  |  | |  |  |
|  | **cg18068240** | | 3 | -72.6 | 151.6 | | 0.8 | 0.15 |
| Whole Blood | *Reading composite score* | |  |  |  | |  |  |
|  | **cg18068240** | | 3 | -64.4 | 135.2 | | 0.72 | 0.11 |
|  | *Math composite score* | |  |  |  | |  |  |
|  | **Age** | | 3 | -64.3 | 135.1 | | 0.15 | 0.1 |
|  | *Writing composite score* | |  |  |  | |  |  |
|  | **TL + cg04111177 + cg04111177*Sex(female) + Volume of Amygdala + Volume of Hippocampus + Sex(female)** | | 8 | -56.8 | 132.9 | | 0 | 0.1 |
|  | *WJ III: Picture Vocabulary* | |  |  |  | |  |  |
|  | **TL + TL*Sex(female) + cg15645634 +Volume of Hippocampus + Volume of Hippocampus*Sex(female) + Sex(female)** | | 8 | -61 | 141.2 | | 0 | 0.2 |
|  | *WASI-II Matrix Reasoning* | |  |  |  | |  |  |
|  | **cg15645634** | | 3 | -72.5 | 151.5 | | 0.67 | 0.1 |
| *Note.* The models passed the test of statistical significance and model diagnostics (for further details, see the Table 5). | | | | | |  |  |  |
